# Determination of pH-dependent antioxidant activity of palm (*Borassus flabellifer*) polyphenol compounds by photoluminol and DPPH methods: a comparison of redox reaction sensitivity

**DOI:** 10.1007/s13205-014-0260-7

**Published:** 2014-10-19

**Authors:** Satyabrata Ghosh, Runu Chakraborty, Utpal Raychaudhuri

**Affiliations:** 1 Department of Ingeniería Rural ETSI Agrónomos, Universidad Politecnica de Madrid, Madrid, Spain; 2 Department of Food Technology and Biochemical Engineering, Jadavpur University, Kolkata, 700032 India

**Keywords:** Antioxidant activity, Antioxidant analyzer, DPPH, Fermentation, Palm juice

## Abstract

Palm juice (*Borassus flabellifer*) is one of the most common and cheap natural juices. Fermented palm juice contains various phytochemical compounds that exhibit antioxidant activity. In the present study, we examined the effects of pH on the production of phytochemicals and their antioxidant activity during the fermentation process. The concentration of total phenolics and flavonoid compounds of fermented palm juice and their antioxidant activity were investigated at various pH. The results showed that total phenolics concentration and antioxidant activity of palm wine and palm vinegar increase as pH increases: 3.5 < 4.5 < 5.5. Maximum flavonoid concentration was obtained at pH 6.5. Measurements of antioxidant activity by conventional DPPH method and Photochem antioxidant analyzer technique were highly correlated, with a corresponding *R* ^2^ value of 0.94.

## Introduction

In recent years, researchers have found that fresh fruits and vegetables significantly contribute to reduction of cardiovascular disease and some types of cancer. Therefore, attempts are being made to correlate epidemiology studies with analysis of normal diets (Ames 1983; Eberthardt et al. 2000; Namiki 1990; Osawa et al. 1990). Fruits and vegetables contain active components including nutrients that contribute to the protection of our body (Dragsted 2003; Hertog et al. 1993; Yang et al. 2001). Antioxidants in food are thought to prevent chronic conditions by preventing damage to important bio-molecules such as DNA, proteins, lipids, etc. (Willcox et al. 2004). Antioxidant compounds are also found in fermented products. In fact, studies have shown that fermentative products are enriched with higher antioxidant activity than their non-fermentative counterparts (Esaki et al. 1997; Lin et al. 2006). Several microorganisms function as natural antioxidant factories (Ishikawa 1992); their antioxidative metabolites (Lin and Yen 1999) produce high free radical scavenging activity (Abe et al. 1998). Yeast is one of these microorganisms that has been shown to increase antioxidant activity of fermented products (Gazi et al. 2001). Yeast produces various enzymes during the fermentation process that have been shown to yield strong antioxidant activity, such as β-glucosidase, carboxyl esterase, feruloyl esterase, etc., (Coghe et al. 2004; Hernandez et al. 2003). *Acetobacter aceti* is another particularly important and commercially viable microorganism in this class. Because this organic compound produces acetic acid, it is commonly used in the production of natural vinegars containing beneficial organic acids, vitamins, phenolic acids, flavonoids and other nutrients showing high antioxidant activity.

The stability and free radical scavenging activity of polyphenol compounds depend on the surrounding pH of the reaction environment (Swiglo and Muzolf 2007). The polyphenol compounds possess various dissociable –OH groups in their chemical structure. It is logical to suspect that the pH of the surrounding medium will influence dissociation rates of the –OH groups in these polyphenol compounds, since previous studies have observed pH influencing the rate of dissociation of oxygen-containing chemical groups in hydroxyflavones and anthocyanins (Muzolf et al. 2008; Lemańska et al. 2001; Borkowski et al. 2005). Free radical scavenging activity would also be expected to change with pH changes. This latter possibility is particularly interesting in the case of palm wine and palm vinegar polyphenol compounds. Palm wine and palm vinegar fermentation is a biological process involving microbes that produce secondary metabolites that are also affected by the pH of the fermenting medium. It is important to study the effects of pH on antioxidant activity in precise experimental settings because of the varying pH environments present during food consumption and digestion. Antioxidant activity could be expected to change as food materials pass through different human body fluids of different pH: pH 1 in the stomach, pH 5.3 in the small intestine, pH 6.8 in mouth saliva, pH 7.4 in blood and tissue fluid, pH 8 in the large intestine, pH 7–8.7 in pancreas, and pH 8.3–9.3 in duodenum (Grzymisławski 2000).

In this study, a new technique for the measurement of antioxidant activity, called Photochem antioxidant analyzer, has been used. This technique is based on a combination of photochemical generation of radicals and chemiluminometric detection. The working principle of this instrument is optical excitation of a photosensitizer substance and subsequent detection of superoxide anion radicals by means of a chemiluminogenic substance (luminol). Data from this antioxidant analyzer were compared with data collected from a conventional 2,2-diphenyl-1-picrylhydrazyl (DPPH) radical scavenging method.

The objective of this work is to monitor the effect of pH on the stability and antioxidant activity of polyphenol compounds in palm wine and palm vinegar produced by fermentation with *Saccharomyces cerevisiae* and *A. aceti*, respectively. The antioxidant activity, measured by a new Photochem technique, is also compared with the conventional DPPH method.

## Materials and methods

### Chemicals

Dextrose, glycerol (GR), KH_2_PO_4,_ K_2_HPO_4_, MgSO_4_·7H_2_O, FeSO_4_·7H_2_O, urea, HPLC grade water, HPLC grade methanol, Folin–Ciocalteu’s phenol reagent, AlCl_3_, and NaNO_2_ were obtained from Merck, India. Yeast extract, peptone, 2,2-diphenyl-1 picrylhydrazyl (DPPH) were obtained from Himedia, India. Antioxidant Analyzer kit was obtained from Analytik Jena, Germany. (+) Catechin hydrate was obtained from Sigma-Aldrich, USA. Gallic acid was obtained from SD Fine Chem Ltd, India.

### Microorganism and culture preparation

#### Yeast and Acetobacter aceti culture preparation

Stock culture of *S. cerevisiae* (NCIM 3045) and *A. aceti* (NCIM 2251) was procured from National Chemical Laboratory (NCL), Pune, India. The culture media of yeast consisted of 0.3 malt extract, 1.0 glucose, 0.3 yeast extract and 0.5 peptone (all in g/100 mL). The organisms were grown at 30 °C and pH 6.5 for an incubation period of 48 h. For *A. aceti*, the media composition was 1.0 tryptone, 1.0 yeast extract, 1.0 glucose, 1.0 calcium carbonate, and 2.0 agar (all in g/100 mL). The organisms were grown at 30 °C and pH 6.0 for an incubation period of 24 h.

### Fermentation media

#### Sample collection

Palm juice (*Borassus flabellifer*) was randomly collected from local traders in rural areas of South 24 Parganas District, West Bengal, India. Traders harvested the palm juice after 12 h of collection in a mud jar through a tapping process using a bamboo tube. After purchase, the bottles of palm juice were kept in a refrigerator. During transportation time (2–3 h), the bottles were carried with ice bags and brought to our laboratory. In the laboratory, the palm juice was preserved at −50 °C in an ultra low temperature freezer (Model C340, New Brunswick Scientific, England).

#### Preparation of fermentation media for wine production

For ethanol fermentation, carbon, nitrogen and other trace elements were added to the palm juice at the appropriate level. The proper composition of fermentation media as described in detail by Ghosh et al. (2012a) was closely followed.

Fermentation was done in a 250 mL flask. 100 mL of fermentation media was taken and the pH was adjusted to 3.5, 4.5, 5.5 and 6.5 before being autoclaved. Then, the media were inoculated with 1 mL yeast culture (concentration of yeast cells in OD was 1.0) and kept at 32 °C for 96 h. The flask was made airtight by paraffin paper to maintain anaerobic conditions. The samples were withdrawn for analysis at designated time intervals with a sterile injection syringe.

#### Preparation of fermentation media for vinegar production

After wine fermentation, sterile sugar (sucrose) was added to the media on the optimized condition and inoculated with 2 mL of an *A. aceti* starter culture solution. The concentration of the *A. aceti* in fermentation media was (1.2 × 10^5^ cells/mL). The temperature and pH were 30 °C and 3.5, 4.5, 5.5 and 6.5, adjusted with 1 N HCl solution and (1:1) aqueous ammonia solution as per experiments at sterile conditions. The incubation time was 96 h and aerobic conditions were maintained by shaking at 150 RPM. The samples were withdrawn for analysis at designated time intervals with a sterile injection syringe (Ghosh et al. 2012b).

## Sample preparation

The fermented sample was withdrawn at appropriate time intervals and then centrifuged at 3,000 RPM for 20 min. The supernatant was collected and filtered with Whatman filter paper no. 1 for the subsequent analytical purpose.

### Alcohol estimation

5 mL of fermented sample was centrifuged (Remi C-24, Mumbai, India) at 3,500 RPM for 10 min. The supernatant solution was used to determine the ethanol concentration by Gas chromatography (Perichrom SGE D11, column BP1-dimethyl polysiloxane).

### Acid estimation

Acetic acid concentration was quantified by an HPLC system (JASCO, MD-2015 Plus Multi wavelength Detector) equipped with absorbance detectors set to 210 nm. The column (ODS-3) was eluted with 0.01 (N) H_2_SO_4_ as a mobile phase, at a flow rate of 0.5 mL/min, and sample injection volume of 20 µL. Standard acetic acid (Merck, India) was used as an external standard.

### Determination of total phenolics content

Using Folin–Ciocalteu (FC) reagent, the total phenolics content (TPC) was measured according to the method (Di Stefano and Guidoni 1989; Singleton et al. 1999). In a spectrophotometer cuvette, an aliquot of 20 µL samples was taken along with 150 µL of Folin–Ciocatlteu reagent, 600 µL of a 15 % Na_2_CO_3_ solution, and a final volume filled to 3,000 µL with distilled water. After 2 h, the increase in absorbance was measured at 784 nm and the concentrations of TPC, expressed as mg/L catechin equivalent (CE), were determined by a calibration curve graph.

### Determination of total flavonoids content

Total flavonoid content (TFC) was measured by aluminum chloride colorimetric assay (Zhishen et al. 1999). An aliquot of 1 mL sample extract or standard solution of catechin was taken in a 10 mL volumetric flask containing 4 mL of distilled water. 0.3 mL of 5 % NaNO_2_ was added to the flask. After 5 min, 0.3 mL of 10 % AlCl_3_ was added. At the 6th min, 2 mL (1 M) NaOH was added and the total volume was then filled to 10 mL with distilled water. The solution was mixed well and the absorbance was measured against a prepared reagent blank at 510 nm. Total flavonoid content was expressed as mg/L catechin equivalent.

### Determination of DPPH radical scavenging activity

The effect of the sample on DPPH radical was estimated according to the procedure described by Brand-Williams et al. (1995). The sample (0.1 mL) was added to 3.9 mL of DPPH (100 μM) in ethanol that was prepared daily. The absorbance was determined at 515 nm after incubation for 45 min. The 0.1 mL ethanol solution and 3.9 mL of DPPH solution were used as control and only ethanol was used as blank. The inhibitory percentage of DPPH was calculated according to the following Eq. (1):

1 $${\text{Scavenging effect }}\left( {\% {\text{ of inhibition}}} \right) \, = \left[ { 1- \, \left( {{\text{absorbance}}_{\text{sample}} /{\text{absorbance}}_{\text{control}} } \right)} \right]\; \times \; 100$$

### Determination of antioxidant activity by photoluminol method

Antioxidant activity was measured by Photochem (Analytik Jena, Germany) using an antioxidant kit (Zhai et al. 2003). The kit was comprised of reagent 1, reagent 2, reagent 3 and reagent 4. Reagent 1 was water, reagent 2 buffer solution, and reagent 3 photoluminating agent. The working solution was prepared by adding 750 µL of reagent 2 to a stock solution of reagent 3. It was mixed well on a vortex and used for further experiment. Reagent 4 was ascorbic acid; its stock solution was prepared by mixing 490 µL of reagent1 and 10 µL 95 % H_2_SO_4_ into a vial containing reagent 4. The resulting solution was mixed well on a vortex for 20–30 s (10 nmol/L). Then, a reagent 4 working solution was prepared by adding 10 µL of reagent 4 stock solution to 990 µL of reagent 1. The blank was prepared with 1.5 mL reagent 1, 1.0 mL reagent 2, and 25 µL reagent 3. The sample solution contained (1.5 − *Y*) mL reagent 1, 1.0 mL reagent 2, and 25 µL reagent 3, where *Y* = 5 µL of sample.

### Statistical analysis

Statistica Release 8 software (Statsoft, USA) was used for data analysis. All experiments were repeated three times and data were presented as mean ± SD for three replications for each sample. The Fisher Least Significance Test was used to check the equality of variances and one-way ANOVA was used to estimate the statistically significant difference (*p* ≤ 0.05).

## Results

The palm juice fermentation occurred through a two-step process: in the first, palm juice was converted to ethanol by *S. cerevisiae*; in the second, acetic acid was produced from ethanol by *A. aceti*. Both the fermentations occurred at optimum temperature and the best nutritional conditions, but variation in pH highly influenced the TPC, TFC and antioxidant activity of palm wine and palm vinegar.

### Effect of pH on TPC, TFC and antioxidant activity in palm wine fermentation

**Fig. 1:**
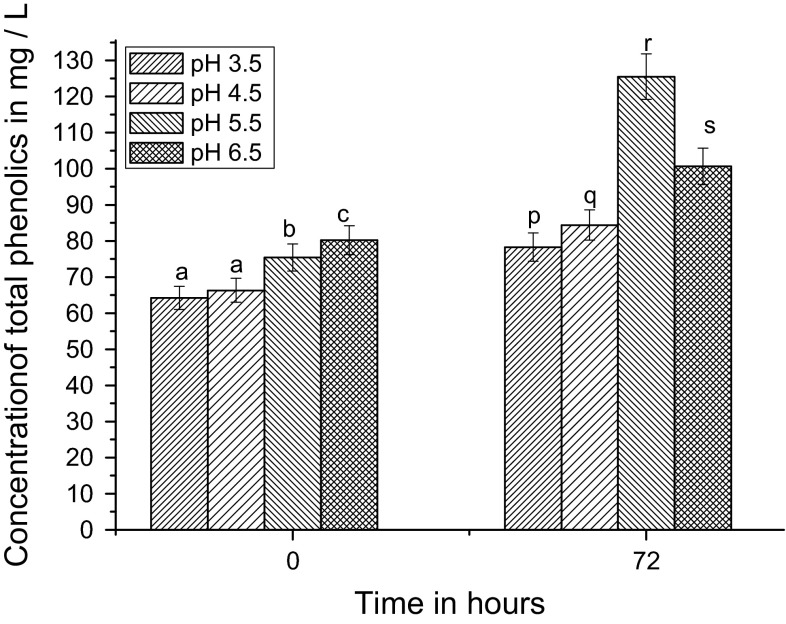
Effect of pH on palm wine fermentation; measurement of total phenolics concentration

pH has a significant impact on optimum yield in the fermentation processes. It also influences the structural stability and antioxidant activity of several phenolic acids and esters in polyphenol compounds. Our results show that the concentration of TPC increased gradually with time during the fermentation process for all pH. At 0 h (before onset of the fermentation process), TPC was highest at pH 6.5, and it was decreased with decreasing order of pH as 3.5 < 4.5 < 5.5 < 6.5. But after 72 h fermentation, the maximum concentration of TPC (125 mg/L) was obtained at pH 5.5. The significance (*p* ≤ 0.05) of these maximum values is depicted in (Fig. 1).

**Fig. 2:**
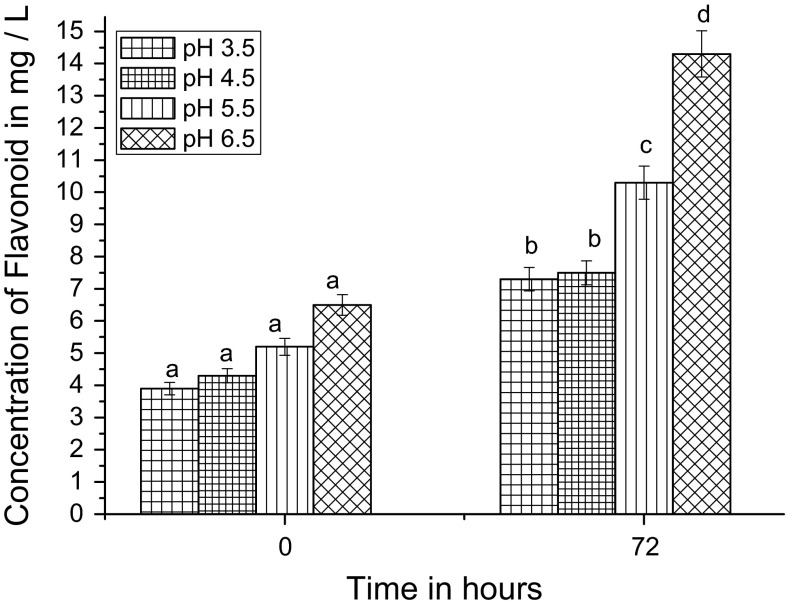
Effect of pH on palm wine fermentation measurement of flavonoid concentration

It has been reported that TFC is more stable at higher pH (Lina et al. 2008). In Fig. 2, we show that at zero hour, the TFC concentration was the maximum at pH 6.5. The maximum value of pH 6.5 was 6.5 mg/L; but this value was not significantly higher than that of the others. After 72 h fermentation, however, TFC was highest at pH 6.5, but the concentration was 14.3 mg/L, significantly higher than the values at other pH.

Our results show that in palm wine fermentation (Table 1), antioxidant activity was increasing with pH up to pH 5.5, but dropped at pH 6.5. At pH 6.5, antioxidant activity was lower than the value of pH 5.5, but higher than values at other pH. The radical scavenging activity test via DPPH and the Photochem method also showed the highest value at pH 5.5: the values were 127.39 (mg/L of CE) and 155 (m/L of AE), respectively. The maximum ethanol concentration was also obtained at pH 5.5 (Table 1). Maximum values of TPC, TFC, antioxidant activity and ethanol concentrations were obtained after 72 h of fermentation, after which time values decreased.

**Table 1:** Qualitative and quantitative data of antioxidant compounds and antioxidant activity of palm wine (after 72 h of fermentation)

pH	Ethanol (g/L)	Total phenol (mg/L)	Total flavonoid (mg/L)	Antioxidant by DPPH (mg/L)	Antioxidant by analyzer (mg/L)
3.5	35.71 ± 5.18^a^	78.3 ± 7.5^e^	7.3 ± 2.7^aa^	91.81 ± 2.8^m^	113 ± 5.4^a^
4.5	48.46 ± 3.84^b^	84.4 ± 5.2^f^	7.5 ± 1.6^aa^	93.33 ± 2.6^m^	120 ± 5.5^b^
5.5	75.49 ± 3.70^d^	125.5 ± 9.9^h^	10.3 ± 1.9^cc^	127.39 ± 3.1^p^	155 ± 6.1^d^
6.5	63.67 ± 3.56^c^	100.7 ± 5.4^g^	14.3 ± 1.7^dd^	106.95 ± 2.5^n^	131 ± 4.1^c^

Values represent mean of triplicates ± standard deviation. Superscript means with different letters are significant different to each other in the same column (*p* = 0.05)

### Effect of pH on TPC, TFC and antioxidant activity in palm vinegar fermentation

**Fig. 3:**
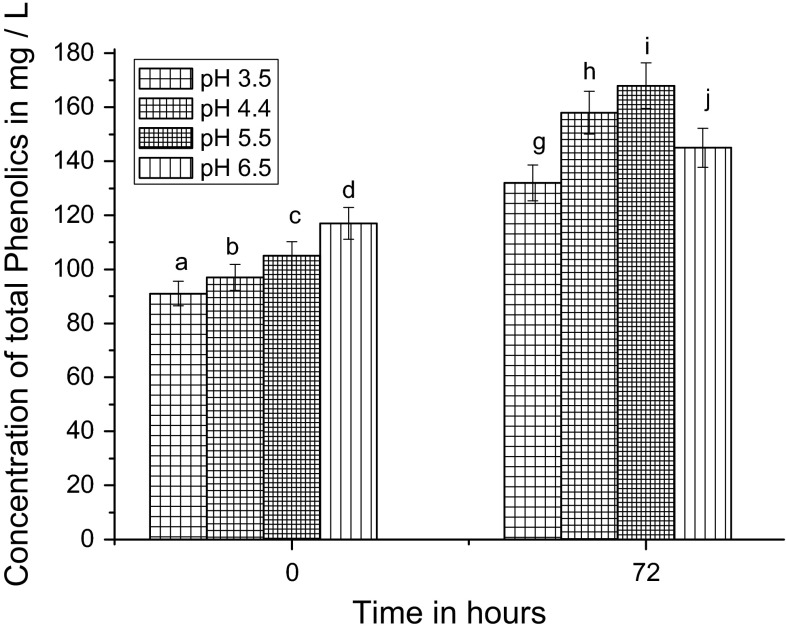
Effect of pH on palm vinegar fermentation; measurement of total phenolics concentration

During acetic acid fermentation, pH influences the biological activity of the *A. aceti*; therefore, optimum pH is considered as the one important factor for producing the highest yield of acetic acid production, as well as the microbial growth. After palm wine fermentation, the pH of the media was adjusted at varying ranges with 1 (N) HCl and 1:1 ammonia solution for acetic acid fermentation. The external pH adjustment at zero hour also reduced antioxidant activity along with TPC and TFC concentrations of the vinegar fermentation media. After the vinegar fermentation started, pH 5.5 was found to be the optimum for highest acetic acid production (68.29 g/L) (Table 2). The TPC concentration was highest at pH 5.5 after 72 h. However, at the initial stage (0 h) of vinegar fermentation, the TPC was significantly higher at pH 6.5 (Fig. 3). But with an increase in time, TPC gradually increased for all pH, with pH 5.5 showing highest concentrations. At pH 5.5, TPC value was 168 mg/L; pH 4.5 was in second position (Fig. 3).

**Table 2:** Qualitative and quantitative data of antioxidant compound and activity for palm juice vinegar produced from palm juice (after 72 h of fermentation)

pH	Acetic acid (g/L)	Total phenol (mg/L)	Total flavonoid (mg/L)	Antioxidant by DPPH (mg/L)	Antioxidant by analyzer (mg/L)
3.5	25.16 ± 7.40^a^	132 ± 4.1^f^	7.2 ± 1.5^ab^	83.22 ± 2.1^r^	78 ± 4.3^k^
4.5	55.73 ± 5.83^b^	158 ± 4.1^g^	7.6 ± 1.8^ab^	89.09 ± 1.2^s^	104 ± 3.4^q^
5.5	68.29 ± 6.27^c^	168 ± 4.2^h^	9.1 ± 1.2^ad^	102.27 ± 2.4^t^	125 ± 5.4^m^
6.5	58.17 ± 5.13^d^	145 ± 4.2^i^	10.7 ± 1.7^bc^	88.84 ± 2.3^u^	93 ± 3.1^n^

Values represent mean of triplicates ± standard deviation. Superscript means with different letters are significant different to each other in the same column (*p* = 0.05)

**Fig. 4:**
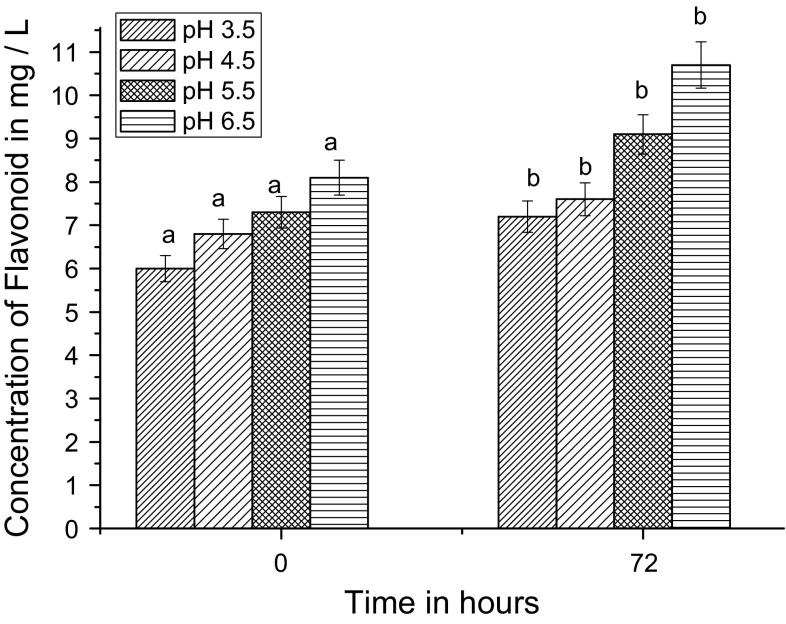
Effect of pH on palm vinegar fermentation; measurement of total flavonoids concentration

Figure 4 shows that at the initial stage (0 h) of fermentation, the TFC of palm vinegar was highest at pH 6.5 (8.1 mg/L). TFC was increased during acetic acid fermentation. After 72 h, the maximum TFC was 10.7 mg/L at pH 6.5; pH 5.5 was second highest for TFC production (9.1 mg/L).

As shown in Table 2, radical scavenging activity of palm vinegar by DPPH and Photochem method was maximized at pH 5.5: the values were 102.27 (mg/L of CE) and 125 (mg/L of AE), respectively. These values were significantly higher (*p* ≤ 0.05) compared to other pH. For palm vinegar, maximum values of TPC, TFC and antioxidant acidity were obtained after 72 h of fermentation and after that all values were decreased with time.

Finally, after comparing the two fermented products (i.e., palm wine and palm vinegar), it can be shown that TPC concentration was higher in palm vinegar but that radical scavenging activity was higher in palm wine (Tables 1, 2).

### Antioxidant activity measurement by Photochem

**Fig. 5:**
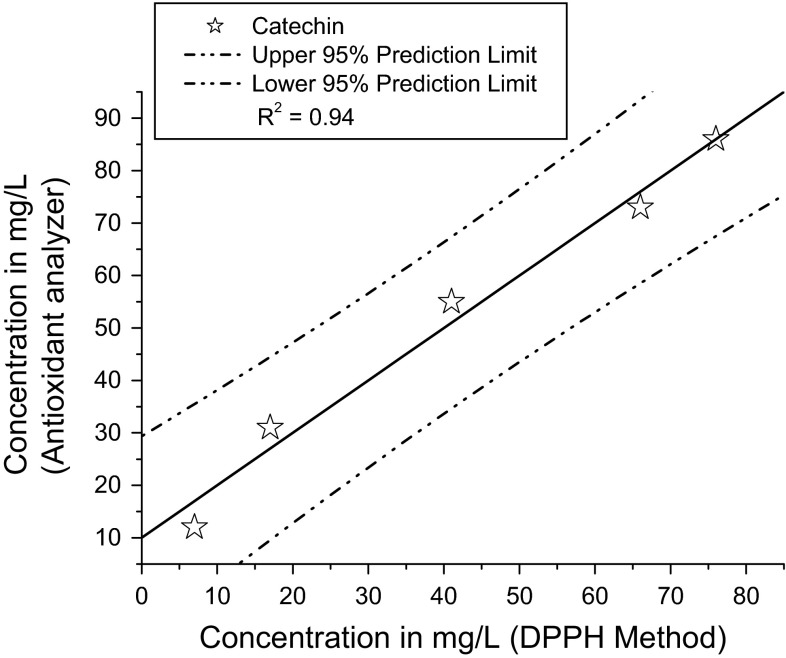
Correlations between antioxidant activities analyzed by DPPH method and by an antioxidant analyzer

A new technique was used for measurement of antioxidant concentration, the Photochem antioxidant analyzer. By this method, the concentration of antioxidant compounds in palm wine was found to be highest at pH 5.5 (155 ± 6.0 mg/L of AE) (Table 1). For palm vinegar, the highest antioxidant concentration was 125 mg/L (of AE) at pH 5.5 (Table 2). The result of the analytical method was validated by linear correlation comparison between the DPPH method and the antioxidant analyzer method. The *R* ^2^ value is 0.94 (Fig. 5).

## Discussion

Palm juice contains various nutrients (i.e., carbohydrate, protein amino acid, ascorbic acid, polyphenol, and flavonoid, etc.) and also shows antioxidant activity. The antioxidant properties are dependent on the pH of the medium, since changes in pKa values correspond to the change of ionization hydroxyl groups or other functional groups of the phenolic compounds (Amorati et al. 2006). Our results showed that the optimum pH for the highest yield of product formation in fermentation corresponded to a maximum concentration of TPC and antioxidant activity. Initially, the 1(N) HCl solution reduced the TPC and TFC concentrations from raw palm juice, and this corresponded to reduced antioxidant activity. The reason for this is that antioxidant activity is influenced by pH in different ways: (1) electrochemical oxidation and H^+^ involvement; (2) the torsion angle of one ring with the rest of the molecules was correlated with scavenging activity due to increased conjugation, which the planarity offers; (3) oxidation stability of the compound; and (4) transformation of the compound (Van Acker et al. 1996; Huang et al. 1996). Earlier researchers reported that antioxidant activity depends upon the oxidation rate of antioxidant compounds, and this oxidation rate was influenced by the surrounding pH (Jovanovic et al. 1994). The catechin showed little antioxidant activity below pH 5, but activity increased above pH 6 (Midori et al. 2001). Physico-chemical properties of phenolics and flavonoid compounds of the raw palm juice should be expected to change due to the external pH, and antioxidant activity should also be expected to reduce (Swiglo and Muzolf 2007). But during palm wine fermentation, yeast growth was affected by varying the pH of the medium: pH 5.5 was optimum for metabolic activation of the yeast, producing the highest volume of metabolites, the highest antioxidant activity, and the maximum content of phenolics and flavonoids.

In the palm vinegar fermentation, the optimal pH for acetic acid production was pH 5.5. *Acetobacter aceti* showed optimal growth in these conditions. Palm vinegar also showed the highest antioxidant activity and TPC after 72 h at pH 5.5. From this observation, it can be concluded that this particular microbe produced a maximum amount of metabolites that are more stable at this pH. But TFC was highest at pH 6.5 in palm vinegar. It means that *A. aceti*-producing TPC is not flavonoid group-containing polyphenol compounds. For both fermentation steps, we have seen that the highest yield of product contained a maximum concentration of antioxidant compounds. This means that the concentration of total phenolics and antioxidant compounds not only depend on pH, but also on the microorganisms’ physiological status. At optimum pH conditions, microorganisms were more metabolically active; therefore, they were able to deliver the highest yield of product along with more secondary metabolites and other substances, which act as antioxidant compounds.

Another important observation was that while concentration of TPC was higher in palm juice vinegar than in palm wine, antioxidant activity was higher in palm wine (Tables 1, 2). The reason behind this is protein: amino acids and other secondary metabolites produced by acetic acid bacteria interfere with the FC (Folin–Ciocalteu) reagent, which are not actual antioxidant compounds (Everette et al. 2010).

Both DPPH and the Photochem method were used for the measurement of concentrations of antioxidant compounds present in the palm wine and palm vinegar by different mechanisms. By linearly correlating these two methods, the *R* ^2^ value was determined to be 0.94. In other words, by measuring the antioxidant concentration with these two methods, we can determine that 94 % of the results are similar in both cases. The very small measured differences were likely due to their different determination mechanism and the different standard antioxidant compound used.

## Conclusions

In our study, we have highlighted the critical observation that pH is the most important factor for controlling photochemical properties of the products of the fermentation process. An optimum pH for a particular fermentation is not always suitable for polyphenol and flavonoid production as well as their stability. During the fermentation process, change in pH has an impact on the oxidative reaction of the phytochemicals. pH 5.5 was found optimal for TPC (125 mg/L) and antioxidant activity (127 mg/L) in palm wine fermentation. pH 5.5 was also optimal for TPC (168 mg/L) and antioxidant activity (102 mg/L) in palm vinegar. But the maximum TFC was found to be at pH 6.5 in both palm wine and palm vinegar fermentations. Whether the individual antioxidant compounds are affected by changes in pH or not during the fermentation are not clear yet and need further research.
